# P-75. Factors Predicting Sequelae After Treatment Of Spondylodiscitis

**DOI:** 10.1093/ofid/ofae631.282

**Published:** 2025-01-29

**Authors:** Fatma Hammami, Khaoula Rekik, Fatma Smaoui, Amal Chakroun, Chakib Marrakchi, Makram Koubaa, Mounir Ben Jemaa

**Affiliations:** Infectious Diseases Department, Hedi Chaker University Hospital, University of Sfax, Tunisia, Sfax, Sfax, Tunisia; Infectious Diseases Department, Hedi Chaker University Hospital, University of Sfax, Tunisia, Sfax, Sfax, Tunisia; Infectious Diseases Department, Hedi Chaker University Hospital, University of Sfax, Tunisia, Sfax, Sfax, Tunisia; Infectious Diseases Department, Hedi Chaker University Hospital, University of Sfax, Tunisia, Sfax, Sfax, Tunisia; Infectious Diseases Department, Hedi Chaker University Hospital, University of Sfax, Tunisia, Sfax, Sfax, Tunisia; Infectious Diseases Department, Hedi Chaker University Hospital, University of Sfax, Tunisia, Sfax, Sfax, Tunisia; Infectious Diseases Department, Hedi Chaker University Hospital, University of Sfax, Tunisia, Sfax, Sfax, Tunisia

## Abstract

**Background:**

The outcome of spondylodiscitis, a serious infection of the intervertebral disc(s) and/or adjacent vertebrae, might be invalidating with the occurrence of sequelae. We aimed to study factors predicting sequelae after treatment of spondylodiscitis.

**Methods:**

We conducted a retrospective study including all patients hospitalized in the infectious diseases department for spondylodiscitis between 2000 and 2022. Tubercular, brucellar and pyogenic spondylodiscitis were included in the study.

**Results:**

We encountered 173 cases, among whom 78 had sequelae (45.1%). No significant difference was noted concerning the age of patients with (54[37-62] years) or without (57[41-67] years) sequelae (p=0.155). Tubercular spondylodiscitis was significantly associated with sequelae (52.6% vs 36.8% ; p=0.038). Anorexia (49.4% vs 31.2% ; p=0.016) and night sweats (57.9% vs 41.9% ; p=0.039) were significantly noted among patients with sequelae. Fever (53.8% vs 64.2% ; p=0.167), back pain (97.4% vs 93.5% ; p=0.295) and asthenia (53.2% vs 44.1% ; p=0.234) were the revealing symptoms among cases with or without sequelae. An accelerated erythrocyte sedimentation rate was noted more frequently among patients with sequelae (90.5% vs 76.5% ; p=0.018). Elevated C-reactive protein level (21.7% vs 20.7% ; p=0.873) and leukocytosis (23.1% vs 24.2% ; p=0.861) were noted among cases with or without sequelae. Treatment duration was significantly longer among patients with sequelae (11[6-15] months vs 7[4-12] months ; p=0.021). Percutaneous abscess drainage was significantly more frequent among patients with sequelae (17.1% vs 6.5% ; p=0.029). Surgery was indicated among patients with or without sequelae (14.3% vs 7.4%), with no significant difference (p=0.141).

**Conclusion:**

The presence of anorexia, night sweats and an accelerated erythrocyte sedimentation rate, especially among tubercular spondylodiscitis cases, were predictor factors of sequelae after treatment. Such patients require a regular follow-up and an adequate treatment duration in order to reduce sequelae.

**Disclosures:**

**All Authors**: No reported disclosures

